# Assessment of Tissue Eosinophilic Infiltration in Invasive Mammary Carcinoma

**DOI:** 10.1155/2024/1514147

**Published:** 2024-09-11

**Authors:** Farah Falah Hasan, Mohammed Haider Fadhil, Zainab Khalid Almukhtar

**Affiliations:** ^1^ Department of Pathology University of Kerbala, Kerbala, Iraq; ^2^ Department of Plastic and Reconstructive Surgery Gazi Al Hariri Teaching Hospital, Baghdad, Iraq; ^3^ Department of Pathology University of Baghdad, Baghdad, Iraq

**Keywords:** HER2-NEU, invasive mammary carcinoma, tissue eosinophilic infiltration

## Abstract

**Background:** Stromal inflammatory cells in malignant tissue have recently gained increasing interest. Unlike the extensive research on tumor-infiltrating lymphocytes, published data about tumor-infiltrating eosinophils in breast cancer are scarce. Furthermore, similar studies have yet to be conducted in Iraq.

**Aims**: The objective of this study is to examine the presence of eosinophilic infiltration by direct visualization using light microscopy and to analyze its relationship with other histological parameters in a group of Iraqi women diagnosed with invasive mammary cancer.

**Methods and material**: A retrospective study enrolled 90 histological samples of invasive mammary carcinoma provided by core needle biopsy from a single center, together with their immunohistochemical results for ER and HER2-NEU. Data reviewing, direct morphological visualizations, and counting eosinophilic infiltration in tissue sections were done by two independent pathologists using light microscopy. The results were statistically correlated with the grade, ER, HER2-NEU, calcification, and axillary lymph node status at presentation.

**Results**: Out of the entire sample size (90), 40 (44%) showed the presence of eosinophilic infiltration in the tissue, both intratumoral and stromal. Further analysis revealed that most eosinophilic infiltrates had an intermediate score (4−19) per 10 consecutive high-power fields. A strong and meaningful statistical relationship was seen between tissue eosinophilic infiltration and HER2/NEU status. A statistically insignificant correlation was seen between tissue eosinophilic infiltration and histological grade, ER receptor status, calcification, and axillary lymph node status at presentation.

**Conclusions**: Eosinophils are tumor-infiltrating cells in breast cancer, both intratumoral and stromal. The presence of tissue eosinophilic infiltration can predict HER2/NEU negativity in breast cancer.

**Summary**

• It is proposed that the assessment of tissue eosinophilia be included in the pathological reporting of invasive mammary cancer, along with its relevance in relation to HER2-NEU status.
• This recommendation is based on the expectation that more substantial cohort studies will support its inclusion.

## 1. Introduction

Invasive mammary carcinoma is the predominant malignancy in females, arising from the terminal duct lobular unit of the breast parenchyma. It is often regarded as a significant primary factor contributing to cancer-related mortality on a global scale. The histological classification is ducal and lobular, and this differentiation does not have any prognostic value [1]. As with other human malignancies, the most accepted theory beyond its pathogenesis is damage in the cellular genetic apparatus by many different triggers [2].

Cancer biology, including development, growth, and progression, is influenced by human immunity [3]. More recently, extensive studies have suggested a possible role of human immunity in chemotherapy response, thereby improving clinical outcomes [4].

The cancer immune cell infiltrates usually consist of T and B lymphocytes, natural killer cells, histiocytes, dendritic cells, neutrophils, eosinophils, and basophils [5]. Eosinophil is a type of granulocyte with bilobed nuclei, large cytoplasmic specific granules, and a characteristic staining pattern of acidophilic dyes. They are vital in parasitic infections and allergic conditions, including immediate-type hypersensitivity reactions [6]. Eosinophils originate from CD34-positive myeloid progenitor cells in bone marrow by stimulation from multiple transcription factors. Then, they are released into the blood and migrate into many body organs and tissues [7]. Eotaxin-1 and C-C motif chemokine ligand (CCL11) are responsible for the recruitment of eosinophils to different tissues and organs through binding to their receptors on the surface of eosinophils, basophil T helper, and airway epithelial cells under the effect of IL-13 and IL-5 stimulation. The physiological functions of eosinophils are complex and still incompletely characterized. They are regarded as multifunctional white blood cells, and they may have a role in tissue remodeling, immunological response to some infectious agents, and functional regulation of cellular immunity [8].

The association between tissue eosinophilic infiltration and human malignancy was founded and reported more than 100 years ago, with a rising conclusion that eosinophils have protumoral and antitumoral effects. However, the mechanisms responsible for their accumulation in the tumor or peripheral blood are still debated [7]. Chemotactic factors, eotaxins, RANTE, and damage-associated molecular patterns (DAMPs) released by necrotic tumor cells are possible eosinophil-attracting agents [5]. It has been found that tumor-associated tissue eosinophilia (TATE) was frequently noticed in malignant lesions, particularly after the application of immune checkpoint inhibitor therapy; they were reported in colorectal, cervical, oral squamous cell carcinoma, Hodgkin's disease, breast, prostate, and ovarian cancer. However, their exact effect on cancer biology is controversial [8]. In most studies and for the most studied tumors, eosinophilic infiltration was recorded to be associated with a better outcome, whether in tumor tissue or in peripheral blood.

Conversely, only a few articles on squamous cell carcinoma showed that TATE is associated with a worse prognosis [9]. The literature is rich in theories regarding the antitumor functions of eosinophils. However, the most accepted theory is that eosinophils act against tumor cells by both direct and indirect mechanisms, the direct mechanism being secretary granule-mediated cytotoxicity. In contrast, the indirect mechanism is immune response modulation by attracting cytotoxic CD8+ T cells [10].

Furthermore, macrophage polarization into M1-like, under the influence of tissue eosinophils, evokes tissue inflammation, promotes phagocytic functions, and promotes the production of hypoxia-inducible factor 1-alpha (HIF1-*α*), which leads to normalization of the vasculature, and facilitates the antitumor effect [11]. Conversely, a protumoral function of eosinophils is facilitated by tumor microenvironment modulation through secretion of metalloproteinase 9 (MMP9), which can promote distant metastasis, the proliferation of fibroblasts and new vesicles formation (angiogenesis), and the production and secretion of multiple growth factors and cytokines [12]. Few studies have shown that eosinophils have no role in the growth of primary tumors; they only induce tumor cell colonization of the metastatic nidus [13].

There is recent evidence suggesting a role of gender in regulating tissue eosinophilia based on the concept that sex hormones (estrogens and/or progesterone) may induce differences in TATE in mammary and gynecological cancer [14]. Except for invasive mammary cancer, in which both intratumoral and stromal eosinophilic infiltration were noted, eosinophils were mainly localized within the tumor stroma [15]. Only scanty data based on both in vitro and in vivo studies focused on TATE in breast cancer was published with conflicting results [16]. Circulating peripheral eosinophilic infiltration was reported in several cancer types, mainly in melanoma and bronchogenic carcinoma, particularly with immunotherapy; less information is known regarding their association with chemotherapy. Accordingly, an association between a better response to immunotherapy and survival has been reported [7]. However, their role in breast cancer and the exact molecular mechanism beyond their accumulation is uncertain and still under investigation [7]. A more recent exciting observation at the “2020 ESMO” congress stated that immune therapy treatment response is associated with an increase in eosinophil gene signature detected in tissue biopsy [17].

Thus, according to literature-based data, it has been concluded that eosinophils either have a positive or a negative impact on the growth of the primary tumor and metastatic potential as well as the antitumor immunotherapy response; its effect depends on tumor type [18], because of the growing emerging idea about the role of sex hormones in influencing and regulating tissue eosinophilic infiltration [7, 19]. In addition, breast cancer is a well-known human malignancy that can express hormone receptors and grow under their influence, as well as its successful story in the era of targeted therapy of human oncology, that is, anti-HER2 immunotherapy. This study is aimed at investigating tissue eosinophilic infiltration, a relatively understudied but intriguing phenomenon among a sample of Iraqi females with breast cancer. The objective was to identify any potential associations that could contribute to improved management strategies or serve as prognostic indicators for this prevalent cancer, both within our society and globally.

## 2. Material and Methods

### 2.1. Study Design and Data Interpretation

This study was conducted in one of Baghdad's private breast center units. It includes oncology, radiology, pathology, and plastic surgery departments. In this study, 90 samples were collected retrospectively from the pathology department in the period from May 2022 to July 2023. Each sample comprises a single histological section of invasive mammary carcinoma stained with hematoxylin and eosin (H&E). These samples were obtained by a core needle biopsy under an ultrasound guide. Additionally, two immunohistochemical sections for ER and Her2NEU were extracted from the laboratory department's archive. Along with these samples, relevant clinical and cytological data were provided. Each sample underwent a thorough review to assign an appropriate grading based on the “Nottingham modification of the Bloom–Richardson system” [20]. Immunohistochemical scoring was also reviewed depending on Allred and IHC scores [21]. The assessment of tissue eosinophilic infiltration was done through direct visual counting of eosinophils in 10 consecutive high-power fields both in the stroma and within the tumor by two independent pathologists using a light microscope (Leica DM500) and then categorized as none: equal to 0; low: equal to 1–4; medium: equal to 5–19; and high: equal to 20 and more [14, 22]. The presence of eosinophilic infiltration in the tissue was statistically correlated with histological grade, ER status, HER2/NEU status, histological calcification, and axillary lymph node status. The axillary lymph node status was determined by fine needle aspiration cytology under ultrasound guidance for lymph nodes that seemed suspicious on radiological imaging at the time of presentation. At our center, we do immunohistochemical staining for ER and HER2-NEU on all core biopsies that were positive for invasive mammary cancer, as required by the oncologist. We use primary antibodies (Dako A/S -Glostrup, Denmark) and an autostainer (X biogenic i6000). Each run was conducted with appropriate positive external controls. In addition, every axillary lymph node that showed signs of abnormality on radiological imaging was examined using a fine needle aspiration technique guided by ultrasonography to confirm the presence of metastases. This study utilized a Leica ICC 50E camera (Leica microsystem/Wetzlar, Germany) to capture photomicrographs.

### 2.2. Inclusion and Exclusion Criteria

In order to avoid bias, we enrolled all consecutive female patients who consulted our centers and underwent ultrasound-guided needle core biopsy as the initial diagnostic approach to obtain tissue for subsequent immunohistochemical staining for ER and HER2-NEU. Only samples with equivocal immunohistochemical results for HER2-NEU, that is, +2, were excluded.

### 2.3. Statistical Analysis

The data analysis used the Statistical Package for Social Science (SPSS) version 25. Frequencies for categorical variables were presented as tables. At the same time, the age range was presented as means ± SD. A comparison between categorical variables was made using the chi-square test. A *p* value of less than 0.05 was regarded as statistically significant, and a *p* value of less than or equal to 0.01 was regarded as highly statistically significant.

### 2.4. Ethics Approval and Consent to Participate

This study was approved by the ethical committee of the Massa Center (reference number 0012-012-14 INT23) on 10/12/2023 (00/12/24) and done by it is an institutional policy in which patient consent was taken primarily at the beginning of consultation for any future retrospective studies.

## 3. Results

**Figure 1:**
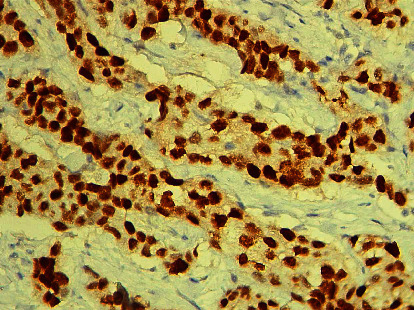
(×100) Immunohistochemically stained section with ER showed a positive nuclear staining pattern.

**Figure 2:**
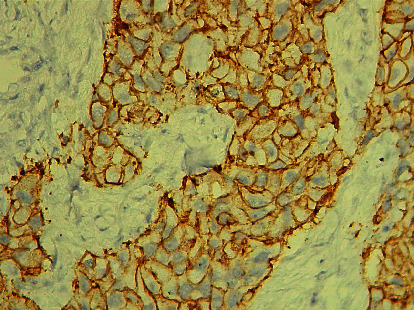
(×100) Immunohistochemically stained section with HER2NEU showed a positive cell membranous staining pattern.

This study enrolled 90 histological samples of invasive mammary carcinoma provided by core biopsy, 54 (60%) from the left breast and 36 (40%) from the right breast. The patient's age range was from 30 to 89 years, with a mean age of 56 ± 12.91 years. A total of 73% were estrogen receptor-positive (Figure 1), and 35.5% were HER2\NEU positive (Figure 2).

**Figure 3:**
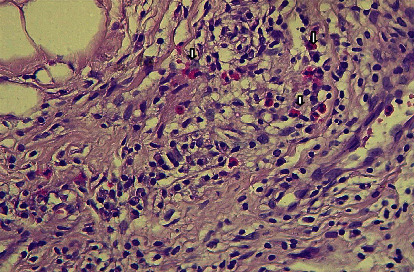
H&E stained histological section showed the presence of stromal eosinophilic infiltration (arrows) in invasive mammary carcinoma.

**Figure 4:**
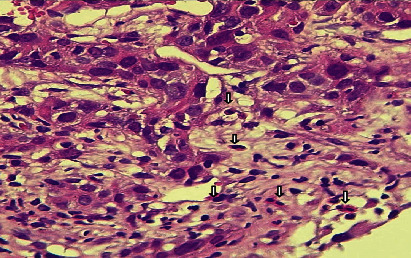
H&E stained histological section showed the presence of intratumoral eosinophilic infiltration (arrows) in invasive mammary carcinoma.

**Figure 5:**
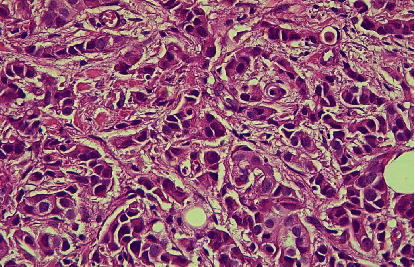
H&E stained histological section showed the absence of tissue eosinophilic infiltration in invasive mammary carcinoma.

Of the total 90 samples, the presence of tissue eosinophilic infiltration, both intratumoral and stromal, was seen in 40 (44%) (Figures 3 and 4); 8 (8%) were scored as high, 82 (91%) were scored as intermediate, and none (0%) were scored as low. However, 50 (55%) of the total sample showed the absence of eosinophilic infiltration (Figure 5).

The distribution of tissue eosinophilic infiltration according to histological grade is illustrated in Table 1.

The correlation of tissue eosinophilic infiltration with histological grade showed a nonsignificant statistical relationship with *p* value 0.133614 (> 0.05) as shown in Table 2.

The correlation of tissue eosinophilic infiltration with hormone receptor status (ER) showed a nonsignificant statistical relationship with *p* value 0.749119 (> 0.05) as shown in Table 3.

The correlation of tissue eosinophilic infiltration with HER2/NEU status showed a highly significant statistical relationship with *p* value 0.000269 (< 0.05) as shown in Table 4.

The correlation of tissue eosinophilic infiltration with tissue calcification showed a nonsignificant statistical relationship with *p* value 0.749119 (> 0.05) as shown in Table 5.

Correlation of tissue eosinophilic infiltration with axillary lymph node status at presentation showed a nonsignificant statistical relationship with *p* value 0.109819 (> 0.05) as shown in Table 6.

## 4. Discussion

Stromal inflammatory cells in malignant tissue have recently gained increasing interest. In contrast to tumor-infiltrating lymphocytes, only very few published data focused on tumor-infiltrating eosinophils in breast cancer, and there were no previous similar Iraqi studies. This study showed that intratumoral and stromal eosinophilia was seen in 44% of the study sample. In this original work, we depended on direct light microscopic visualization of eosinophils on H&E-stained histological sections without immunohistochemistry or molecular technique. Eosinophils have a characteristic apparent morphology (bilobated nucleus and very characteristic intensely acidophilic granules), allowing easy detection. Two pathologists did morphological visualization and counting to minimize interobserver variation. Cost-effective analysis has become increasingly important in the medical field in general and histopathology in particular. It should be appreciated, encouraged, and agitated in the researcher's mind to apply this policy primarily when feasible in their initial work, investigating any new idea, particularly in a resource-limited setting. On the other hand, we thought that direct visual counting of intact viable eosinophils on H&E sections is easier and more effective than that with the aid of immunohistochemistry to overcome any artifact produced by background staining due to eosinophil degradation and degranulation, a common phenomenon recorded in malignant tumors [23].

For better clarification of the results and due to the initial observation that some heterogeneity in the density of tissue eosinophilic infiltration was always observed within the same core and in different cores belonging to the same patient, a score for counting eosinophils was applied depending on some previous studies on tissue eosinophilic infiltration within malignant tumors in general [14, 22]. Accordingly, this study showed that the vast majority of eosinophilic infiltration (91%) was within the intermediate density score (i.e., 5–19 per 10 consecutive high-power fields). Only 8% were scored as high, that is, more than 20 per 10 consecutive high power fields, and 0% fell within the low score, that is, 0–4 per 10 consecutive high power fields. Therefore, for statistical consideration to avoid bias, when we correlate with other variables, we regarded all samples showing eosinophilic infiltration as a positive variable regardless of their score. The only shared data among invasive mammary cancers with high tissue eosinophil counts is HER2/NEU negativity, which was noted in all samples. A surprising result was obtained from a previous study by Rose-Marie et al. [24], which found that eosinophils are not tumor-infiltrating cells in breast cancer. However, their work on searching for eosinophils was achieved using “microarray analysis.” A “microarray” is a paraffin block that contains numerous tiny-sized representative tissue spots from hundreds of different cases of interest, assembled on a single slide, allowing rapid analysis of multiple small tissue samples while minimizing technical variation in the results, a manner utterly different from our work in which the searching was done by scanning all the available tissue materials, which often include a minimum of three cores per patient, a policy done at our center. Furthermore, intratumoral heterogeneity for tissue eosinophilic infiltration, an observation obtained in our study and agreed with other studies [16, 25], when present, is regarded as an impediment to the use of this sort of analysis. Chouliaras et al., when investigating tissue eosinophilia in their work through RNA sequencing data for eosinophil signatures in mammary cancer by the “CIBERSORT” technique, found that “TATE,” both intratumoral and stromal, was present in only 3.7% of the cases, mostly in luminal type [15]. A larger sample size and a completely different manner of research clarified this disagreement with our results.

This study found that the correlation of tissue eosinophilia with histological grade (degree of differentiation) showed a nonsignificant statistical relationship, which agreed with another previous study on tissue eosinophilia in breast cancer done by Grisaru-Tal et al. [16]. The present study also concludes that there is a nonsignificant statistical relationship between eosinophilic infiltration and ER receptor status; a significant correlation was only obtained with HER2-NEU status. These results disagreed with Grisaru-Tal et al. [16]. The use of microarray analysis, immunohistochemical visualization of eosinophils with the limitation above, and a completely different method for interpreting the results (scanned photomicrographs for analysis) all explain any disagreement that may have occurred with our results.

The breast cancer data set published by the “Royal College of Pathologists” has stated that assessment of PR receptor status in breast cancer is optional, primarily due to its uncertain predictive value in adjuvant therapy when compared with ER expression. That is the reason for the exclusion of PR receptor status from our work. Furthermore, according to literature-based data, almost all ER-negative, PR-positive breast cancer is rare [26].

This study showed a nonsignificant statistical relationship between tissue eosinophilic infiltration and tumoral calcification. Unlike coronary atherosclerosis, in which eosinophils are considered a new player for calcification [27], they have no such effect on mammary cancer. We also found a nonsignificant statistical relationship between tissue eosinophilia and axillary lymph node status at presentation. Unfortunately, no published data with the same aim regarding calcification and axillary lymph node status is available for comparison. Thus, based on our data, tissue eosinophilic infiltration does not correlate with tumor grade and axillary lymph node metastasis, which are both regarded as powerful prognostic factors in breast cancer [21].

This study found that the only significant association of tissue eosinophilic infiltration was achieved with HER2-NEU negativity. This finding, if confirmed in the future, means that the presence of tumor-associated tissue eosinophilic infiltration can predict negativity for HER2-NEU, minimizing the subsequent use of immunohistochemistry and allowing for more cost-effective rapid initiation of the treatment protocol in certain situations and resource-limited settings. On the other hand, this idea may benefit quality control assurance.

Advances in understanding the interplay between cellular constituents of the tumor microenvironment in breast cancer have shown that eosinophils and T lymphocytes cooperate to stimulate therapy responses to immune checkpoint inhibitors [17]. A recent study done by Ghebeh et al. [28] observed that there is a systemic accumulation of eosinophils in breast cancer patients who respond to treatment with nivolumab, an anti-PDL1 drug.

Blomberg et al. [29] found that intratumoral expression of eosinophil signature-containing genes increased in responders to immune checkpoint inhibitor therapy. These observations uncovered the role of eosinophils in modulating the effect of immunotherapy in patients with breast cancer, enhancing their therapeutic benefit. PDL1 expression on eosinophils is polarized toward the cell membrane region in contact with T cells, of interest; CD8+ T-cells express higher levels of PDL1 than CD4+ T-cells. The main mechanism of eosinophil-mediated interaction is through enhancing the activation of CD8+ T-cells by making a direct contact with them through PDL1. [17–29]. Intratumoral eosinophil recruitment was directed by CD4+ T cells, IL-5, and IL-33 [30].

The establishment of eosinophil-based therapeutic strategies necessitates extensive prospective clinical trials in order to improve new therapeutic avenues, particularly in the era of the application of PDL1 immunotherapy in triple-negative breast cancer. Thus, our original results on tissue eosinophilic infiltration in breast cancer warrant further extensive work and discussion. We hope this study is the starting point toward awareness of tissue eosinophilic infiltration in breast cancer among Iraqi patients with a need for standardization in pathological work regarding this concept.

### 4.1. Limitation

The main limitation of this study was lack of the relevant clinical information, as with any retrospective study. Another discussion-related limitation was the scarcity of published data with a similar aim for comparison and extensive discussion as with any original study.

## 5. Conclusion

Eosinophils are tumor-infiltrating cells in breast cancer, both intratumoral and stromal. There is a significant statistical relationship between the presence of tissue eosinophilic infiltration and HER2-NEU negativity. In contrast, a nonsignificant statistical relationship was achieved between tissue eosinophilic infiltration with histological grade, ER receptor status, calcification, and axillary lymph node status at presentation.

### 5.1. Recommendation

A proposal for including assessment of tissue eosinophilic infiltration during pathological reporting of invasive mammary carcinoma with the incorporation of its significance regarding HER2-NEU status is hopefully advised, definitely after further large-scale prospective clinicopathological studies searching for any associated peripheral eosinophilia as well as tumor-infiltrating lymphocytes with a particular concern to the era of immune checkpoint inhibitors, an issue of most significant concern in modern oncology.

## Figures and Tables

**Table 1:** The distribution of tissue eosinophilic infiltration according to histological grade.

**Histological grade**	**Tissue eosinophils**		**Total**
	**Presence**	**Absence**	
Low-grade GI	10	20	30
Intermediate grade GII	22	16	38
High-grade GIII	8	14	22
Total	40	50	90

**Table 2:** Correlation of tissue eosinophilic infiltration with histological grade.

**Histological grade**	**Tissue eosinophils**		**Total**	**p** ** value**
	**Presence**	**Absence**		
Well-differentiated GI	10	20	30	0.133614
Moderately to poorly differentiated GII and GIII	30	30	60	
Total	40	50	90	

**Table 3:** Correlation of tissue eosinophilic infiltration with hormone receptor (ER) status.

**ER receptor status**	**Tissue eosinophils**		**Total**	**p** ** value**
	**Presence**	**Absence**		
Positive	30	36	66	0.749119
Negative	10	14	24	
Total	40	50	90	

**Table 4:** Correlation of tissue eosinophilic infiltration with HER2/NEU status.

**HER2/NEU status**	**Tissue eosinophils**		**Total**	**p** ** value**
	**Presence**	**Absence**		
Positive	6	26	32	0.000269
Negative	34	24	58	
Total	40	50	90	

**Table 5:** Correlation of tissue eosinophilic infiltration with tissue calcification.

**Tissue calcification**	**Tissue eosinophils**		**Total**	**p** ** value**
	**Presence**	**Absence**		
Positive	10	14	24	0.749119
Negative	30	36	66	
Total	40	50	90	

**Table 6:** Correlation of tissue eosinophilic infiltration with axillary lymph node status proven by fine needle aspiration cytology at presentation.

**Axillary lymph node status**	**Tissue eosinophils**		**Total**	**p** ** value**
	**Presence**	**Absence**		
Positive	14	10	24	0.109819
Negative	26	40	66	
Total	40	50	90	

## Data Availability

The data used to support the findings of this study are available from the corresponding author upon request.
